# A Novel Mutation of the Calcium-Sensing Receptor Gene Causing Familial Hypocalciuric Hypercalcemia Complicates Medical Followup after Roux-en-Y Gastric Bypass: A Case Report and a Summary of Mutations Found in the Same Hospital Laboratory

**DOI:** 10.1155/2019/9468252

**Published:** 2019-02-13

**Authors:** Elin Rebecka Carlsson, Mai-Britt Toft Nielsen, Anne Mette Høgh, Rikke Veggerby Grønlund, Mogens Fenger, Louise Ambye

**Affiliations:** ^1^Department of Clinical Biochemistry, Copenhagen University Hospital Hvidovre, Denmark; ^2^Department of Endocrinology, Copenhagen University Hospital Hvidovre, Denmark; ^3^Gubra, Hørsholm, Denmark

## Abstract

Heterozygous inactivating mutations in the calcium-sensing receptor (CaSR) gene are known to cause familial hypocalciuric hypercalcemia (FHH), usually a benign form of hypercalcemia without symptoms of a disrupted calcium homeostasis. FHH can be mistaken for the more common primary hyperparathyroidism (PHPT), for which surgical treatment may be needed. We describe a case of a 36-year-old woman with hypercalcemia and elevated PTH, initially suspected of having PHPT. Sequencing of the CaSR-gene revealed a mutation in nucleotide 437, changing the amino acid in position 146 from Glycine to Aspartate. The mutation was previously undescribed in the literature, but a very low calcium:creatinine clearance ratio supported the diagnosis FHH. A few years later, the patient's two daughters were tested and the association between mutation and hypercalcemia could be confirmed. The patient was gastric bypass-operated and therefore, due to malabsorption and increased risk of fracture, was in need of adequate calcium supplementation. The chronically elevated calcium levels challenged medical followup, as calcium sufficiency could not be monitored in a traditional manner. Eventually the patient developed elevated alkaline phosphatase, a further increased PTH and a decreased DXA T-score indicating calcium deficiency and bone resorption. As a supplement, all CaSR-mutations found at our hospital, 2005-2018.

## 1. Introduction

Calcium homeostasis is crucial for many biological functions and is regulated by direct and indirect effects of parathyrine (PTH) on kidney, bone, and intestine, released as a reaction to sensing of hypocalcemia in the parathyroid gland, mediated through the calcium-sensing receptor (CaSR) [1]. Calcium is usually absorbed throughout the whole intestine, where an adequate vitamin D-level is required for optimal uptake [2]. If the supply is insufficient, increased PTH stimulates urinary calcium reabsorption and resorption of calcium from the skeleton. In addition, through their own calcium-sensing receptors, the kidneys promote urinary calcium reabsorption independently of PTH [1].

Heterozygous inactivating mutations in the CaSR-gene are known to cause familial hypocalciuric hypercalcemia (FHH), also called benign hypercalcemia due to its mild, often asymptomatic phenotype [3, 4]. Neonatal severe hyperparathyroidism and autosomal dominant hypocalcemia (ADH) are other more severe clinical manifestations of mutations in the same gene. So far, more than 130 FHH-causing mutations of the CaSR-gene have been described and novel mutations keep being reported [1].

In patients with hypercalcemia, the main reason for sequencing the CaSR-gene in search for mutations is to distinguish patients with FHH from patients with primary hyperparathyroidism (PHPT), who otherwise have a similar presentation biochemically [4, 5]. Parathyroidectomy, a removal of one or several of the parathyroid glands is needed to treat symptomatic PHPT that typically arises as a sporadic mutation in an adenoma [6], while FHH rarely requires treatment and cannot be cured surgically, unless a total parathyroidectomy is performed [7]. Most patients with FHH have a mutation in the CaSR-gene (FHH 1) but occasionally the cause is a mutation in two other genes, GNA11 (FHH2) or AP2S1 (FHH3), the latter associated with a more severe phenotype [1, 5, 8, 9]. A similar clinical presentation in patients without mutations related to the CaSR-gene may also be caused by CaSR-blocking autoantibodies, as seen in autoimmune hypocalciuric hypercalcemia (AHH) [3]. Apart from genetic sequencing, the calcium:creatinine clearance ratio is important in distinguishing between PHPT and FHH [10].

Calcium deficiency and secondary hyperparathyroidism are also problematic and commonly seen in patients treated for obesity with Roux-en-Y gastric bypass, where a part of the stomach and proximal intestine is excluded from food passage [11]. These patients are at increased risk of getting a fracture compared to the general population [12] and an adequate supplementation of calcium and vitamin D, monitored by measurements of calcium-ion, 25-hydroxy-vitamin D, and PTH, is important to reduce this risk [13].

We describe a case of a 36-year-old gastric bypass-operated woman with hypercalcemia and elevated PTH. Sequencing of the CaSR-gene revealed a previously undescribed mutation in nucleotide 437, changing the amino acid in position 146 from Glycine to Aspartate. Six years later, her two daughters were tested. One of the daughters was found to carry the same mutation.

## 2. Case Presentation

A 36-year-old woman was referred to the department of endocrinology for further examination of hypercalcemia, which was discovered during routine blood tests after gastric bypass operation 1 year earlier. There was no history of kidney stones, fractures, or osteoporosis that may be a result of hypercalcemia, and she had no known hyperthyroidism, Addison's disease, malignancy, sarcoidosis, or any other granulomatous disease that could explain the hypercalcemia. She had lost contact with her mother and sister, her only living relatives. Thus, a family history of hypercalcemia could not be investigated.

The patient inconsistently took calcium and vitamin D3 supplements in addition to iron, cobalamine, and multivitamins after the gastric bypass operation. She did not take thiazide diuretic or any other medications. She had symptoms of depression, anxiety, and tiredness and was later prescribed antidepressant medication. She also had recurrent episodes of dizziness, tremor, sweating, and fatigue, which resolved with the ingestion of carbohydrate and was related to hypoglycemia. Reactive hypoglycemia is a known late complication of gastric bypass operation induced by inappropriate hyperinsulinemia after the intake of rapidly absorbed carbohydrates [13]. The reactive hypoglycemia responded to dietitian instructions.

Repeated blood tests showed Ca-ion between 1.42 and 1.47 mmol/l (ref: 1.18 – 1.32 mmol/l), PTH between 6.3 and 8.9 pmol/l (ref: 1.7 – 7.1 pmol/l), and 25-hydroxy vitamin D between 43 and 58 nmol/l (ref: > 50 pmol/l). Alkaline phosphatase and thyroid function were normal. Dual-energy X-ray absorptiometry (DXA) showed T= -0.6 and T= -0.2 at the lumbar spine and total hip, respectively (Table 1).

Based on the mild hypercalcemia and the high normal to slightly elevated PTH, the patient was suspected of having primary PHPT. Before referral to a surgeon, FHH, the rare differential diagnosis to PHPT, had to be excluded. Genetic testing for mutations in the CaSR-gene showed a heterozygous mutation in nucleotide 437, changing the amino acid in position 146 from Glycine (G) to Aspartate (D) (c.[437G>A];[=], p.[(G146D)];[=]), reference sequence NM_000388.3 OMIM 601199), here called G146D. The found mutation was not formerly associated with FHH. However, urine calcium:creatinine clearance ratio was very low, 0.0029 and 0.0017 on two occasions, which verified the diagnosis of FHH, at the same time excluding the more common PHPT.

The CaSR is a member of the subfamily C of G-protein-coupled receptors which have seven transmembrane domains and function as disulfide-linked homodimers [14]. Its gene is located on chromosome 3q13.3-21.1 [15] and includes seven exons where exon 2-7 are protein coding [1]. The mutation G146D is located in exon three, in the coding region of the receptor's extracellular Venus flytrap domain, more specifically as a part of LB1, a domain important for the ligand binding of calcium ions (Ca^2+^) and the amino acid L-tryptophan (L-Trp) that functions as an allosteric activator, enhancing the sensitivity of the receptor towards Ca^2+^ [14]. The binding of Ca^2+^ at this position seems to be important for maintaining the structural integrity of the receptor whereas other binding positions for Ca^2+^ are of more importance for receptor activity. Mutations in amino acids positioned as number 145 and 147, flanking number 146 on each side, have both been shown to eliminate the Ca^2+^ induced receptor activity. Based on this and the fact that the amino acid in position 146 is a part of the L-Trp binding cleft, it is likely that a mutation in position 146 reduces the receptor activity. Indeed, predictions from public* in silico* evaluation tools (Mutation Taster, PolyPhen, Provean) that were used to assist in the interpretation of the DNA-variant G146D all predicted a homozygous mutation G146D to be disease causing when factors like the location of the mutation regarding change of splice site, active site, or amino acid, as well as the nucleotide conservation between species, were taken into consideration. This knowledge about the CaSR-structure and consequence of mutations in neighboring amino acid positions was however not available at the time the patient was diagnosed.

Six years later, the patient's two daughters, 12 and 17 years old, were tested for the mutation. The youngest daughter did not have any mutation in the CaSR-gene, but she had a low level of D-vitamin associated with secondary hyperparathyroidism, low Ca-ion and elevated alkaline phosphatase. The eldest daughter was found to be carrying the same mutation in the CaSR-gene as her mother, associated with hypercalcemia and inappropriately high PTH. She, too, had a low level of D-vitamin. Both daughters had normal thyroid and liver function tests. The patient at this point had an unchanged mild hypercalcemia, but additionally she had developed elevated alkaline phosphatase, a further increased PTH-level and a decreased DXA T-score (lumbar spine) indicating FHH accompanied by calcium deficiency and bone resorption (Table 1).

## 3. Discussion

We have described a case story of a woman with hypercalcemia and permanently elevated PTH, where sequencing of the CaSR-gene revealed a probable disease-causing mutation in nucleotide 437, changing the amino acid in position 146 from Glycine to Aspartate. Mutations in the same locus had not been previously described, but the clinical presentation with a low calcium:creatinine clearance strongly supported the diagnosis FHH. Six years later, following CaSR-gene sequencing in two daughters, we were finally able to confirm that the novel mutation found most likely is the cause of the hypocalciuric hypercalcemic phenotype in this family. At this time, updated mutation databases and a better understanding of CaSR-structure and function were helpful in predicting the mutation to be disease causing, inactivating the CaSR.

In hyperparathyroidism, a correct diagnosis is important for several reasons. For our patient, the diagnosis of FHH1 ensured that she avoided unnecessary parathyroidectomy and finally enabled prescription of the proper vitamin and mineral supplementations. For the eldest daughter, who is a carrier of the same mutation, the diagnosis meant that she, like her mother, was not considered for surgery. In general, it is important to test females of FHH families, because calcium gets across the placenta and, in FHH mothers, leads to fetal hypercalcemia with a subsequent suppression of fetal parathyroid function resulting in the risk of transient, neonatal, hypocalcemic tetanus [16]. Apart from this, FHH is usually asymptomatic with rarely seen complications such as pancreatitis, gallstones, or chondrocalcinosis [3, 4, 10]. As complications are correlated to the degree of hypercalcemia, most distinctly in patients with FHH3 [1], simply monitoring the calcium level without treatment often suffice. Extreme values or symptoms of hypercalcemia might be treated with calcimimetics [17].

Chronic hypercalcemia is associated with kidney stones and osteoporosis (the latter not in FHH), but also neuromuscular and psychiatric symptoms [6]. The patient in the case vignette reported fatigue and depression, which could be caused by hypercalcemia. To our knowledge, this is the first case report of a patient with FHH treated for obesity with gastric bypass surgery, a combination that challenges medical investigation and followup. Especially, monitoring of calcium homeostasis needs a broader approach, as plasma levels of calcium and PTH are chronically elevated. In this case, malabsorption after gastric bypass surgery resulting in calcium deficiency could be identified by an increased level of PTH, compared to the habitual level of PTH, eventually combined with an increased level of alkaline phosphatase and a reduced bone mineral density (BMD). This complexity emphasizes the importance of qualified medical followup after gastric bypass surgery.

Interestingly, this case report also illustrates another complexity, namely, the large variation within the spectrum of hyperparathyroidism, here with several variants seen in the same family. First, we have our patient with FHH and calcium deficiency, second the eldest daughter with FHH and normal level of PTH, and last the youngest daughter with secondary hyperparathyroidism due to deficiency of vitamin D and calcium. In common, all family members have low levels of D-vitamin.

Compared to the relatively high prevalence of PHPT, FHH is rare [4]. At our hospital, we started in 2005 to perform genetic testing by polymerase chain reaction (PCR) and Sanger sequencing of the six protein coding exons (exon 2-7) and intron boundaries of the CaSR-gene. Since the beginning, we have sequenced DNA from 822 patients suspected of having a parathyroid adenoma. Altogether, we have found 56 different mutations in heterozygous form (Supplementary Table S1), a majority of which as of September 2018 had been described in the medical literature [5, 18–26] and/or reported in mutation databases like ClinVar [27]. Most of the mutations found were inactivating mutations. In total, a mutation was found in 91 (11%) of the patients tested. The real mutation prevalence in this population is, although, most certainly higher. This is due to the limitations of the method that only sequence the coding exons and the intron boundaries of the CaSR-gene but not the promoter region, the deep intron regions, and the 3′untranslated region. Moreover, large genomic deletions and sequence variants in primer binding sites can be missed, resulting in false-negative test results. Mutations in other genes, for example, GNA11 or AP2S1 where mutations can result in FHH2 and FHH3, are not examined.

Important to note and as illustrated in this case report is that the interpretation of the clinical consequence of the mutation can change depending on the data available for a found mutation at the given time. The clinical picture, supported by results from routine biochemical analyses and diagnostic imaging, will always be of great value in diagnosis.

## Figures and Tables

**Table 1:** Results from biochemical analysis and measures of bone mineral density.

		I		II_1_	II_2_
CaSR mutation		+/-		+/-	-/-

		at presentation	at time for II_1_'s and II_2_'s testing	at diagnosis	at testing

Age		36	42	17	12

Biochemical analyses:	Reference:				

25-hydroxy vitamin D (nmol/L)	> 50	53	40 ↓	25 ↓	24 ↓

Calcium ion, ionized (mmol/L)	1.18 – 1.32	1.47 ↑	1.46 ↑	1.45 ↑	1.15

Parathyrine (pmol/L)	1.1 – 7.1	8.9 ↑	10.9 ↑	4.2	14.1 ↑

Alkaline Phosphatase (U/L)	35 - 105^a^	89	118 ↑	91	213

Calcium:Creatinine clearance ratio	> 0.010	< 0.003 ↓	not measured		

Measure of bone mineral density:		Result	Result		

DXA Lumbar spine (T-score)	> - 1.0	-0.6	-1.2 ↓		

DXA Total hip (T-score)	> - 1.0	-0.2	0.1		

For the patient (I), results are shown at presentation, as well as at the time for testing of her two daughters (II_1_ and II_2_) whose results at testing are shown. Apart from CaSR-gene sequencing and results from I's presentation, all blood samples from all three patients are drawn and analyzed at the same day.

^a^Reference range for Alkaline Phosphatase differs with age; the one for adults is shown in the table, for the girl aged 17 years reference range is 42-102, and for the girl aged 12 it is 143-396. ↑ indicates that the result is above upper reference limit. ↓ indicates that the result is below lower reference limit. +/-, heterozygous mutation in the CaSR-gene; -/-, no mutation in the CaSR-gene.

## Supplementary Materials

Supplementary Materials Supplementary Table S1. A summary of all CaSR-mutations found at our hospital laboratory between 2005 and 2018, their associated phenotypes (based on available laboratory results), in silico predictions, and references to the literature.

## References

[1] Hannan F. M., Babinsky V. N., Thakker R. V. (2016). Disorders of the calcium-sensing receptor and partner proteins: Insights into the molecular basis of calcium homeostasis. *Molecular Endocrinology*.

[2] Wasserman R. H. (2005). Vitamin D and the intestinal absorption of calcium: a view and overview. *Vitamin D*.

[3] Hannan F. M., Thakker R. V. (2013). Calcium-sensing receptor (CaSR) mutations and disorders of calcium, electrolyte and water metabolism. *Best Practice & Research Clinical Endocrinology & Metabolism*.

[4] Christensen S. E., Nissen P. H., Vestergaard P., Mosekilde L. (2011). Familial hypocalciuric hypercalcaemia: a review. *Current Opinion in Endocrinology & Diabetes and Obesity*.

[5] Vargas-Poussou R., Mansour-Hendili L., Baron S. (2016). Familial hypocalciuric hypercalcemia types 1 and 3 and primary hyperparathyroidism: similarities and differences. *The Journal of Clinical Endocrinology & Metabolism*.

[6] Fraser W. D. (2009). Hyperparathyroidism. *The Lancet*.

[7] Shinall M., Dahir K., Broome J. (2013). Differentiating familial hypocalciuric hypercalcemia from primary hyperparathyroidism. *Endocrine Practice*.

[8] Marx S. J. (2018). Familial hypocalciuric hypercalcemia as an atypical form of primary hyperparathyroidism. *Journal of Bone and Mineral Research*.

[9] Hovden S., Rejnmark L., Ladefoged S. A., Nissen P. H. (2017). AP2S1 and GNA11 mutations - not a common cause of familial hypocalciuric hypercalcemia. *European Journal of Endocrinology*.

[10] Heath D. A. (2000). Familial hypocalciuric hypercalcemia. *Reviews in Endocrine and Metabolic Disorders*.

[11] Hewitt S., Søvik T. T., Aasheim E. T. (2013). Secondary hyperparathyroidism, vitamin D sufficiency, and serum calcium 5 years after gastric bypass and duodenal switch. *Obesity Surgery*.

[12] Nakamura K. M., Haglind E. G. C., Clowes J. A. (2014). Fracture risk following bariatric surgery: a population-based study. *Osteoporosis International*.

[13] Mingrone G., Bornstein S., Le Roux C. W. (2018). Optimisation of follow-up after metabolic surgery. *The Lancet Diabetes & Endocrinology*.

[14] Geng Y., Mosyak L., Kurinov I. (2016). Structural mechanism of ligand activation in human calcium-sensing receptor. *ELife*.

[15] Janicic N., Soliman E., Pausova Z. (1995). Mapping of the calcium-sensing receptor gene (CASR) to human chromosome 3q13.3-21 by fluorescence in situ hybridization, and localization to rat chromosome 11 and mouse chromosome 16. *Mammalian Genome*.

[16] Thomas A. K., McVie R., Levine S. N. (1999). Disorders of maternal calcium metabolism implicated by abnormal calcium metabolism in the neonate. *American Journal of Perinatology*.

[17] Marx S. J. (2017). Calcimimetic use in familial hypocalciuric hypercalcemia—a perspective in endocrinology. *The Journal of Clinical Endocrinology & Metabolism*.

[18] Pidasheva S., Canaff L., Simonds W. F., Marx S. J., Hendy G. N. (2005). Impaired cotranslational processing of the calcium-sensing receptor due to signal peptide missense mutations in familial hypocalciuric hypercalcemia. *Human Molecular Genetics*.

[19] Nissen P. H., Christensen S. E., Ladefoged S. A., Brixen K., Heickendorff L., Mosekilde L. (2012). Identification of rare and frequent variants of the CASR gene by high-resolution melting. *Clinica Chimica Acta*.

[20] Nissen P. H., Christensen S. E., Heickendorff L., Brixen K., Mosekilde L. (2007). Molecular genetic analysis of the calcium sensing receptor gene in patients clinically suspected to have familial hypocalciuric hypercalcemia: phenotypic variation and mutation spectrum in a danish population. *The Journal of Clinical Endocrinology & Metabolism*.

[21] Rasmussen A. Q., Jørgensen N. R., Schwarz P. (2018). Identification and functional characterization of a novel mutation in the human calcium-sensing receptor that co-segregates with autosomal-dominant hypocalcemia. *Front Endocrinol (Lausanne)*.

[22] Guarnieri V., Canaff L., Yun F. H. J. (2010). Calcium-sensing receptor (CASR) mutations in hypercalcemic states: studies from a single endocrine clinic over three years. *The Journal of Clinical Endocrinology & Metabolism*.

[23] Fukumoto S., Chikatsu N., Okazaki R. (2001). Inactivating mutations of calcium-sensing receptor results in parathyroid lipohyperplasia. *Diagnostic Molecular Pathology*.

[24] Frank-Raue K., Leidig-Bruckner G., Haag C. (2011). Inactivating calcium-sensing receptor mutations in patients with primary hyperparathyroidism. *Clinical Endocrinology*.

[25] Leech C., Lohse P., Stanojevic V., Lechner A., Göke B., Spitzweg C. (2006). Identification of a novel inactivating R465Q mutation of the calcium-sensing receptor. *Biochemical and Biophysical Research Communications*.

[26] Hannan F. M., Nesbit M. A., Zhang C. (2012). Identification of 70 calcium-sensing receptor mutations in hyper- and hypo-calcaemic patients: evidence for clustering of extracellular domain mutations at calcium-binding sites. *Human Molecular Genetics*.

[27] Landrum M. J., Lee J. M., Benson M. (2016). ClinVar: public archive of interpretations of clinically relevant variants. *Nucleic Acids Research*.

