# Supplementary material for: A Novel Mutation of the Calcium-Sensing Receptor Gene Causing Familial Hypocalciuric Hypercalcemia Complicates Medical Followup after Roux-en-Y Gastric Bypass: A Case Report and a Summary of Mutations Found in the Same Hospital Laboratory
Source: Case Rep Endocrinol. 2019 Feb 13;2019:9468252. doi: 10.1155/2019/9468252 (PMC6393866; doi:10.1155/2019/9468252)
Supplement: Supplementary Materials — Supplementary Table S1. A summary of all CaSR-mutations found at our hospital laboratory between 2005 and 2018, their associated phenotypes (based on available laboratory results), in silico predictions, and references to the literature. [file 9468252.f1.pdf]

Supplementary table S1. All mutations found at our hospital laboratory between 2005-2018, their associated phenotypes, in silico predictions and references to the literature as of September 2018.

EXON MUTATIONS

Nukleotide change	Amino acid change	Number of patients (if less than all, number with available lab data)	Clin Var	Mutation Taster	Medical literature	Phenotype
**nonsynonymous substitutions**						
c.40 A>G	T14A	1	NA	probably harmless	benign (1)	PHPT
c.55G>A	A19T	1	another mutation in this aa is likely benign	disease causing		PHPT
c.92A>G	D31G	1	NA	disease causing		FHH
c.188A>G	Y63C	1	NA	disease causing		FHH
c.207G>A	R69H	1	likely pathogenic	disease causing	other mutation affecting the same aa found in patient with FHH (2)	FHH
c.221T>C	M74T	1	-	disease causing	novel	FHH[u]
c.242T>C	I81T	1 (0)	-	disease causing	found in patient with FHH (3)	
c.310T>G	V104P	1	-	disease causing	novel, but other mutation affecting the same aa found in patient with ADH (2)	FHH
c.377A>T	D126V	3 (2)	-	disease causing	recently described (4)	ADH
c.437G>A	G146D	2	-	disease causing	novel	FHH[u]
c.513C>A	S171R	1	uncertain significance	disease causing	found in patient with FHH (5) other mutation affecting the same aa cause disease (6)	FHH
c.644A>G	D215G	1	another mutation in this aa is likely pathogenic	disease causing		FHH
c.658C>G	R220W	4 (1)	NA		found in patients with FHH (2,7)	FHH
c.691G>A	E231K	2	-	disease causing	novel	ADH
c.741_742insT	D248*	1	-	disease causing	novel	FHH[u]
c.748G>A	E250K	2(1)	likely benign	probably harmless	benign (8), FHH (2)	normal
c.848T>C	I283T	1(0)	conflicting interpretations	disease causing	Found in patient with PHPT (9)	
c.862A>G	I288V	1	-	disease causing	found in patient with FHH (2)	FHH[u]
c.903_903delC	S302Pfs*	4	-	disease causing	novel	FHH[u]
c.1015C>T	P339S	1	-	disease causing	novel	FHH
c.1056G>A	W352*	3	-	disease causing	found in several patients with clinical signs of FHH (6)	FHH[u]
c.1189G>A	G397R	2	uncertain significance	disease causing	found in several families with FHH (6)	FHH/atypical phenotype
c.1253A>G	Y418C	1	-	disease causing	novel	FHH
c.1283C>A, c.492+5T>A	A428D, intron	1	-	disease causing	novel	FHH
c.1389C>A	H463Q	1	NA	disease causing		PHPT/FHH
c.1393C>T	R465W	1	uncertain significance	disease causing	other mutation affecting the same aa cause disease (10)	FHH
c.1406T>G	F469C	1	NA	disease causing		FHH light
c.1479C>A	N493K	1	NA	disease causing		PHPT
c.1525G>A	G509R	1	pathogenic	disease causing	found in family with FHH (6)	FHH
c.1663A>G	I555V	1	NA	disease causing	found in patient with FHH (6)	FHH[u]
c.1680C>G, c.1681T>G	C561G, T560T	1(0)	-	disease causing	novel	
c.1837G>A	G613R	1	likely pathogenic	disease causing		FHH
c.1970C>A	S657Y	1	-	disease causing	novel	FHH
c.2039G>A[a]	R680H	1	uncertain significance	disease causing	other mutation affecting the same aa found in patient with NSHPT (2)	FHH[u]

Nukleotide change	Amino acid change	Number of patients	Clin Var	Mutation Taster	Medical literature	Phenotype
c.2089G>A	V697M	1	-	disease causing	novel	FHH
c.2224T>C	W742R	1	-	disease causing	found in patient with FHH (6)	FHH
c.2345T>G	L782R	1	-	disease causing	novel	FHH
c.2383_2383delC	R795Gfs*	1(0)	-	disease causing	found in family with FHH (6)	
c.2607C>G[a]	I869M	1	-	disease causing	novel	FHH light[u]
c.2649_ 2650insGCT	A884dup	1	-	disease causing	novel	FHH
c.2785A>G	R929G	1	-	probably harmless	novel	PHPT[u]
c.2956G>T	A986S	1	other - variant	probably harmless	a frequent variant (2)	PHPT
c.3220A>G	N1074D	1	NA	disease causing		normal
**synonymous substitutions**						
c.78 C>G	A26A	2	benign	disease causing		PHPT/SHPT
c.114T>C	F38F	1	probably benign	disease causing		normal
c.537A>G	E179E	1	benign	disease causing		FHH[u]
c.573G>A	E191E	3	likely benign	disease causing	found in PHPT patient (9)	PHPT
c.819C>G	G273G	1	-	disease causing	novel	FHH light
c.906C>T[a]	S302S	1(0)	-	disease causing	novel	
c.1338T>C	N446N	1	NA	disease causing		PHPT[u]
c.2064C>T	F688F	1(0)	likely benign	disease causing	found in reference population (9)	
c.2610G>A	E870E	17(16) +3[a]	benign	disease causing	found in reference population (9)	PHPT/normal

INTRON MUTATIONS

Nukleotide change	Number of patients	Clin Var		Human Splicing Finder	Phenotype[a]
c.1608+2T>A	1	-	disease causing	change of splicesite, frameshift	FHH[u]
c.492+57G>A	1	-	probably harmless	probably not affecting gene splicing	PHPT
c.-9 G>A	1	NA	probably harmless	-	PHPT[u]
c.186-69G>A	1	-	disease causing	possible change of splice site	FHH
c.186-64G>A	1	-	probably harmless	possible change of splice site	FHH light
c.1733-4A>G	1	-	disease causing	probably not affecting gene splicing	PHPT

Total Numbers:

Different mutations:	58
Patients with a mutation:	91
Patients sequenced:	822

Legend to Supplementary Table S1.

Table show all CASR-mutations found in in our laboratory between year 2005 and September 2018. Nukleotide- as well as amino acid changes are shown. The nomenclature is based on the reference sequence NM_000388.3; the nucleotide numbers starts with the A of the ATG translation-initiation site as base 1.

The phenotype-column indicates the most probable phenotype based on available lab results in the current laboratory software system (containing data from 2009). Laboratory data were not available for all patients as some of them are external patients from other Hospital Regions and others might have been diagnosed before 2009. If lab data were not avilable for all patients with a certain mutaion, the number of patients with available lab data is shown in paranthesis in the number of patients column. Urinary calcium:creatinine clearance ratio was available for some patients, marked with a [u].
It is important to note, that phenotype categorization might not be the same as the final clinical diagnosis, as only biochemical data and no clinical information as for example anamnestic data, family history or results from parathyroid scintigraphy have been used in preparing this table.

Several different in silico tools not shown in table have been used at the time for analysis and interpretation of each found mutation. Here, in silico interpretations from Mutation taster (www.mutationtaster.org) as of September 2018 are shown as an example, as well as information from the ClinVar archive (https://www.ncbi.nlm.nih.gov/clinvar/) and references to peer reviewed papers in international journals. The intron mutations are also evaluated through Human splicing finder (http://www.umd.be/HSF3/index.html). In the material, we see several conflicts between in silico interpretations and the patient phenotype, especially Mutation taster is restrictive with excluding known variants from being possibly disease causing, as it demands observations of all three genotypes (heterozygous, homozygous and wild-type) in unaffected individuals, in order to exclude a damaging effect of the allele. This complexity illustrates the importance of clinical and laboratory investigation.

Abbreviations: aa, aminoacid; ADH, Autosomal dominant hypocalcemia; FHH, Familial hypocalciuric hypercalcemia; FHH light, a "light" version of FHH with normal to mildly elevated plasma calcium and normal PTH (parathyroid hormone); normal, normocalcemia and normal PTH; NA, information not available; NSHPT, Neonatal severe primary hyperparathyroidism; PHPT, Primary hyper parathyroidism; SHPT, secondary hyper parathyroidism. [a] Three of the patients with other mutations also had a coincidental mutation E870E.

References:

1. Pidasheva S, Canaff L, Simonds WF, Marx SJ, Hendy GN. Impaired cotranslational processing of the calcium-sensing receptor due to signal peptide missense mutations in familial hypocalciuric hypercalcemia. Hum Mol Genet. 2005;14(12):1679–90.
2. Hannan FM, Nesbit MA, Zhang C, Cranston T, Curley AJ, Harding B, et al. Identification of 70 calcium-sensing receptor mutations in hyper- and hypo-calcaemic patients: Evidence for clustering of extracellular domain mutations at calcium-binding sites. Hum Mol Genet. 2012;21(12):2768–78.
3. Nissen PH, Christensen SE, Ladefoged SA, Brixen K, Heickendorff L, Mosekilde L. Identification of rare and frequent variants of the CASR gene by high-resolution melting. Clin Chim Acta. Elsevier B.V.; 2012;413(5–6):605–11.
4. Rasmussen AQ, Jørgensen NR, Schwarz P. Identification and Functional Characterization of a Novel Mutation in the Human Calcium-Sensing Receptor That Co-Segregates With Autosomal-Dominant Hypocalcemia. Front Endocrinol (Lausanne). 2018 Apr 25;9(April):1–10.
5. Vargas-Poussou R, Mansour-Hendili L, Baron S, Bertocchio JP, Travers C, Simian C, et al. Familial hypocalciuric hypercalcemia types 1 and 3 and primary hyperparathyroidism: Similarities and differences. J Clin Endocrinol Metab. 2016;
6. Nissen PH, Christensen SE, Heickendorff L, Brixen K, Mosekilde L. Molecular genetic analysis of the calcium sensing receptor gene in patients clinically suspected to have familial hypocalciuric hypercalcemia: phenotypic variation and mutation spectrum in a Danish population. J Clin Endocrinol Metab. 2007;92(11):4373–9.
7. Guarnieri V, Canaff L, Yun FHJ, Scillitani A, Battista C, Muscarella LA, et al. Calcium-Sensing Receptor (CASR) mutations in hypercalcemic states: Studies from a single endocrine clinic over three years. J Clin Endocrinol Metab. 2010;95(4):1819–29.
8. Fukumoto S, Chikatsu N, Okazaki R, Takeuchi Y, Tamura Y, Murakami T, et al. Inactivating mutations of calcium-sensing receptor results in parathyroid lipohyperplasia. Diagnostic Mol Pathol. 2001;10(4):242–7.
9. Frank-Raue K, Leidig-Bruckner G, Haag C, Schulze E, Lorenz A, Schmitz-Winnenthal H, et al. Inactivating calcium-sensing receptor mutations in patients with primary hyperparathyroidism. Clin Endocrinol (Oxf). 2011;75(1):50–5.
10. Leech C, Lohse P, Stanojevic V, Lechner A, Göke B, Spitzweg C. Identification of a novel inactivating R465Q mutation of the calcium-sensing receptor. Biochem Biophys Res Commun. 2006;342(3):996–1002.
